# Pictorial size perception in schizophrenia

**DOI:** 10.1186/s41155-021-00201-z

**Published:** 2021-11-21

**Authors:** Maria Lúcia de Bustamante Simas, Ana Cristina Taunay Maranhão, Aline Mendes Lacerda, Flora Silva Teixeira, Carlos Henrique Resende Freire, Cecília Coimbra da Silva Raposo, Georgia Mônica Marque de Menezes

**Affiliations:** Laboratório de Percepção Visual, Programa da Pós Graduação em Psicologia, Centro de Filosofia e Ciências Humanas, Universidade Federal de Pernambuco, Recife, Brazil

**Keywords:** Schizophrenia, Psychosis, Spatial perception, Size perception, Pictorial perception

## Abstract

In this study, we compared visual pictorial size perception between healthy volunteers (CG) and an experimental group (EG) of people diagnosed with schizophrenia. We have been using paintings by Salvador Dalí and Rorschach plates to estimate visual pictorial size perception. In this transversal, ex post facto, and quasi-experimental study, we observed differences between EG and CG. Schizophrenic in-patients perceived sizes about 1.3-fold greater than healthy volunteers (*p*=0.006), implying that pictorial size perception is altered in some way in schizophrenia. Considering the present and previous results, this measurement of diameter size of first pictorial perception may be a useful estimate of some aspects of perceptual alterations that may be associated with psychotic symptoms in prodromal and acute schizophrenic episodes and other related mental states. Eventually, this may help in preventing people from evolving to acute episodes.

## Introduction

Although evidence that sensory processes are altered in schizophrenic patients is a widely accepted finding, the exact nature and impact of such alterations are not fully understood. For instance, how is perception, behavior, and cognition of a psychotic mind affected by the salience of a bright ray of sunlight passing through? Or by a sudden shift in noise? Or by the salience of all white waving bed cover sheets pending on windows subjected to a windy surround? Or even, by the glimpse of everyday images of objects, shades, videos, photos, people, and the like? Or by rushing thoughts, and images, old and recent memories, talks, noises, and all of these in an extreme emotional state? Evidence currently in the literature does not have ready answers to those questions.

Studies on size perception (Davis et al., 1967), perceptual organization (Silverstein & Keane, 2011), illusions (King et al., 2017), contrast sensitivity and spatial frequency (Flevaris et al., 2014; Graham & Meng, 2011; Green et al., 2008; McBain et al., 2010; Shoshina et al., 2015; Silverstein et al., 2017), face perception and recognition (Kim et al., 2015; Marosi et al., 2019; McBrain et al., 2010; Silverstein et al., 2014; Vakhrusheva et al., 2014), visual integration (Postmes, et al., 2014; Silverstein, et al., 2009; Silverstein, et al., 2012; Silverstein et al., 2015), visual search (Chen, 2011; Dias et al., 2013), and mismatch negativity or odd ball paradigm (Avissar et al., 2018; Hamilton et al., 2019), among others, have all addressed issues related to sensory processes in schizophrenia from a basic research point of view.

The only works indirectly related to our study we found in the literature were about size perception of the Müller-Lyer and Ponzo illusions (King et al., 2017; Pessoa et al., 2008; Shoshina et al., 2011; Weckowics & Witney, 1960), or about altered pictorial perception, in this case, the perception of pareidolias, in Lewis-body dementia and/or Parkinson disease (Mamiyaet al., 2016; Uchiyama et al., 2012; Uchiyama et al., 2015; Yoko et al., 2014). We briefly review those.

Weckowics and Witney (1960) found that schizophrenics showed larger Müller-Lyer illusion effects than controls, but their group of non-schizophrenic psychiatric patients also provided responses consistent with the perception of larger magnitude effects than controls but, in turn, lower magnitudes than schizophrenics. They could not explain those latter results. In addition, in 2008, Pessoa et al.. suggested the use of the Müller-Lyer illusion for screening schizophrenic patients based on an animal model.

Shoshina et al. (2011) conducted studies on the Ponzo and the Müller-Lyer illusions with schizophrenic patients and non-psychiatric “healthy” volunteers. On one hand, they found opposite results for schizophrenics either with less or more than 10 years of illness. The former group showed lower sensitivity to Ponzo illusion than healthy patients, whereas the opposite was true for those patients with more than 10 years after the diagnosis of schizophrenia. On the other hand, they found that schizophrenic patients with more than 10 years of illness were more susceptible to the Müller-Lyer illusion than those with less than 10 years of illness, who in turn were more susceptible than healthy patients. However, they found gender differences. Apparently, male schizophrenic patients are less susceptible to the Ponzo illusion than schizophrenic females.

The present study focuses on the actual perception of the psychotic afflicted mind, it does not focus on illusions. We originally started our studies centered around the visual perception of the world during prodrome, acute, or long-term psychotic episodes. We argue that schizophrenic patients present disrupted pictorial size perception showing a tendency towards the perception of large magnitudes compatible with high sensitivity to extremely low fundamental spatial frequencies (mediating form and pictorial perception). Such a hypothesis arises from the empirical observation that from the first-person narrative point of view, during acute psychotic episodes, schizophrenics report perceiving pareidolia-like images (PLI) of huge magnitudes, in natural scenes, or at least, perceived as much larger than the actual size of the referred object.

**Fig. 1:**
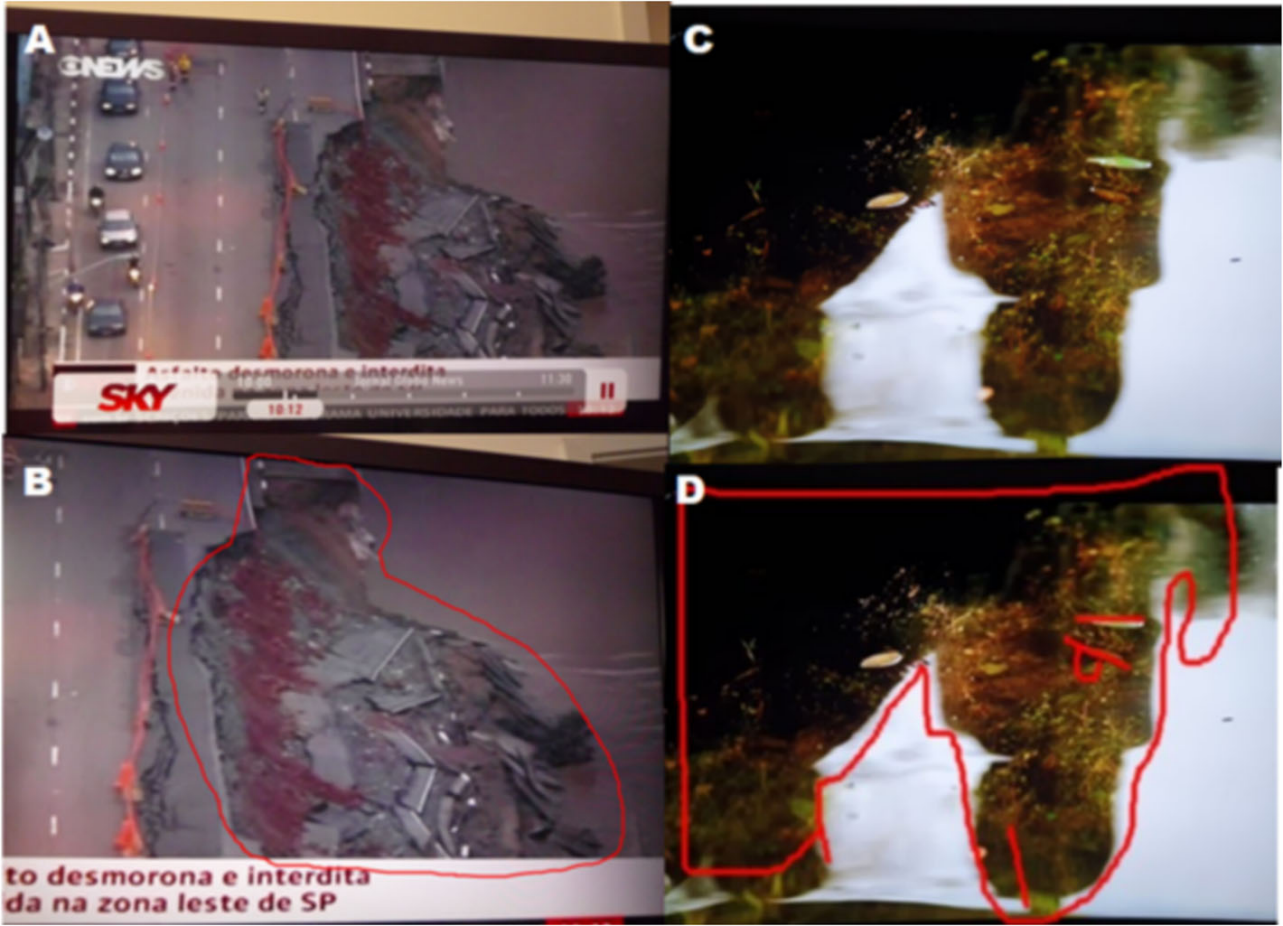
On the left, the pareidolia of a “beggar or homeless” (**B**) is perceived in large size in a highway landslide (**A**); on the right, the reflexion of two people talking (**C**) is an assembled pareidolia-like image of a horse’s head (**D**)

Here, we named PLI those images spontaneously perceived in natural everyday scenes deriving from the assembly of bits and pieces of the natural observed scene put together, in huge magnitudes, and pictured as meaningful or meaningless objects. Examples of PLI are shown in Fig. 1 where a highway landslide (Fig. 1 A) appears to be a huge “beggar or homeless” (Fig. 1 B) and where a reflection of two people talking (Fig. 1 C) appears to be the huge head of a horse (Fig. 1 D).

In our approach, we have been using images for the observation of PLI that are not filtered like those of Uchiyama et al. (2012, 2015; Mamiya et al., 2016; Suzuki et al., 2017; Yoko et al., 2014) for dementia with Lewy bodies (DLB), as well as for Parkinson disease. In their view, the perception of pareidolia itself is the key point of the investigation. Also, most of the images used in these studies to induce the perception of pareidolia adopted low-pass-filtered images of visual scenes as stimuli. In our case, we used some unfiltered paintings by Salvador Dalí and the *magnitude represented by the diameter* of the perceived PLI is the major issue under investigation. The choice of paintings by Salvador Dali to estimate image pictorial size perception is related to the great variety of sizes and PLI within a single painting, present in many of his works. For comparison purposes, we also used Rorschach test plates, exclusively measuring the diameter of the pictorial perception within a delimitated area of the first at glance perceived image, with the aim of illustrating the occurrence of the effect under investigation, regardless of the general configuration of the pictures.

In our early studies (Lacerda et al., 2020; Simas et al., 2011), we have used a selection of 24 paintings by Dalí and asked people with schizophrenia and healthy volunteers to point out the first at glance image they perceived in each of those paintings. We were able to reproduce these results with different small sample populations of schizophrenic patients repeatedly from 2002 to 2012. Altogether, we have used this procedure with over 100 experimental and 100 healthy volunteers (Lacerda et al., 2020; Simas et al., 2011) and have consistently observed that schizophrenics perceive larger images than controls and that different populations of schizophrenics appear to perceive progressively larger images that apparently related to the severity of the prodromal or acute/chronic states. In this early work (Lacerda et al., 2020; Simas et al., 2011), we used the concept of “concatenation of forms” in a way that was analogous to the concepts of PLI that we now adopt.

This study reports experiments run in a clinic and a hospital comparing in-patients to healthy volunteers with the aim of replicating previous results (Lacerda et al., 2020; Simas et al., 2011) even though using a selection of only 10 Dalí paintings and 10 Rorschach plates. Volunteers encircled, by themselves, the first at glance perceived image within each of the paintings, or plates, directly on the iPad2 display.

## Method

### Study settings

Experiments were conducted at “Hospital Ulysses Pernambucano, HUP,” “Clínica Santo Antônio de Pádua,” public school “Escola Maria da Conceição do Rêgo Barros Lacerda” at Recife, Pernambuco, Brazil, and at “Serviço de Atenção Psicológica da UFSC (SAPSI).” Volunteers of the former two were in the Experimental Group, EG, and those of the latter two, in the control group, CG. The trial was approved by the Institutional Review Board (Comitê de Ética em Pesquisa, CEP) at the Federal University of Pernambuco, UFPE, (Trial Registration Number: CAAE n°15479213.5.0000.5208) and volunteer patients (or legal guardians) as well as controls read and signed consent forms agreeing to participate in the experiment and were free to leave the study at any time. This work was consistent with the principles outlined in the internationally recognized standards for the ethical conduct of human research.

### Experimental group (EG)

Fifty-nine patients diagnosed with schizophrenia (34 men, 25 women) participated in the study (mean age = 34.7 ±12.9 years old). Table 1 shows the occurrence of ICDs in the sample. The inclusion criteria were (i) age 18–60 years old; (ii) minimum four years of formal education and compatible Mini-Mental State Examination, MMSE, scores (≥10); (iii) F20 CID-10 diagnostic; and (iv) at least a week duration of continuous medication, whereas the exclusion criteria were (i) patients with ocular diseases, (ii) with organic cerebral diseases, and (iii) excessive agitation or aggressivity. Formal education of experimental volunteers was characterized by 66.67% high school level, 30.55% incomplete fundamental, and 2.78% omitted this information.

**Table 1:** Characterization of EG per diagnostics

ICD - diagnostics	*N* = 59
F20.0	40
F20.1	6
F20.2	1
F20.5	1
F20.9	2
F29	5
Others (F19, F25, F23)	4
Total	59

Regarding medications, 43.13% of the volunteers used typical antipsychotics, 11.37% risperidone, and 2.37% other atypical antipsychotics. Note that medication was given in combination with other drugs being 18.48% mood stabilizers, 12.3% antihistamines, 3.79% anxiolytics, and 8.53% other medications including antidepressants. The great majority of these patients were/are regular smokers.

### Control group (CG)

Fifty-nine healthy volunteers (mean age = 26.44 ±9.18 years old), students, and workers from public schools or universities were selected for the CG (24 men, 35 women). The inclusion criteria were (i) 18–60 years old and (ii) low, middle, and high levels of formal education, while the exclusion criteria were (i) ocular diseases, (ii) familial history of neuropsychiatric disorders, (iii) drug or alcohol abuse, and (iv) last eventual use of alcohol for a period shorter than 24 h. Formal education of control volunteers was characterized by 85.42% high school level and 14.58% incomplete fundamental. Table 2 shows the characteristics of each sample. We were unable to equate age in both groups. The EG is older than the CG. Gender was also unequal in numbers but this did not affect our results as seen in previous studies (Lacerda et al., 2020).

**Table 2:** Characterization of the EG and CG in terms of age, gender, and formal education

	CG (*N* = 59)	EG (*N* = 59)	*p* value
Age (mean, SD)	26.44 ±9.18	34.12 ±8.39	*p* = 0.00*
Gender	24 male, 35 female	34 male, 25 female	
Education level	85.42% high school, 14.58% incomplete fundamental	66.67% high school, 30.55% incomplete fundamental, 2.78% no response	

*Mann-Whitney *U* test

### Instruments and equipment

Instruments used with the EG consisted of medical records, consent forms, and sociodemographic and familial history interviews, as well as the Brazilian validated versions of Mini-Mental State Examination (MMSE) and the Scale of Positive and Negative Symptoms (PANNS), as well as the Rasquim E Acuity Chart. These same instruments were used for the CG, except for medical records and PANNS.

We used paintings selected from previous studies (Lacerda et al., 2020; Simas et al., 2011) originally carried out with printed photos of 24 Dali paintings (10 × 15 cm). Thus, ten 10 × 15-cm digital photos of paintings by Salvador Dali were used together with 10 × 15-cm digital photo of the ten Rorschach plates (from Rorschach test) for inspection by the volunteers and posterior comparison of sizes of the encircled image seen at first glance, regardless of any attribution of meaning by the observer.

**Fig. 2:**
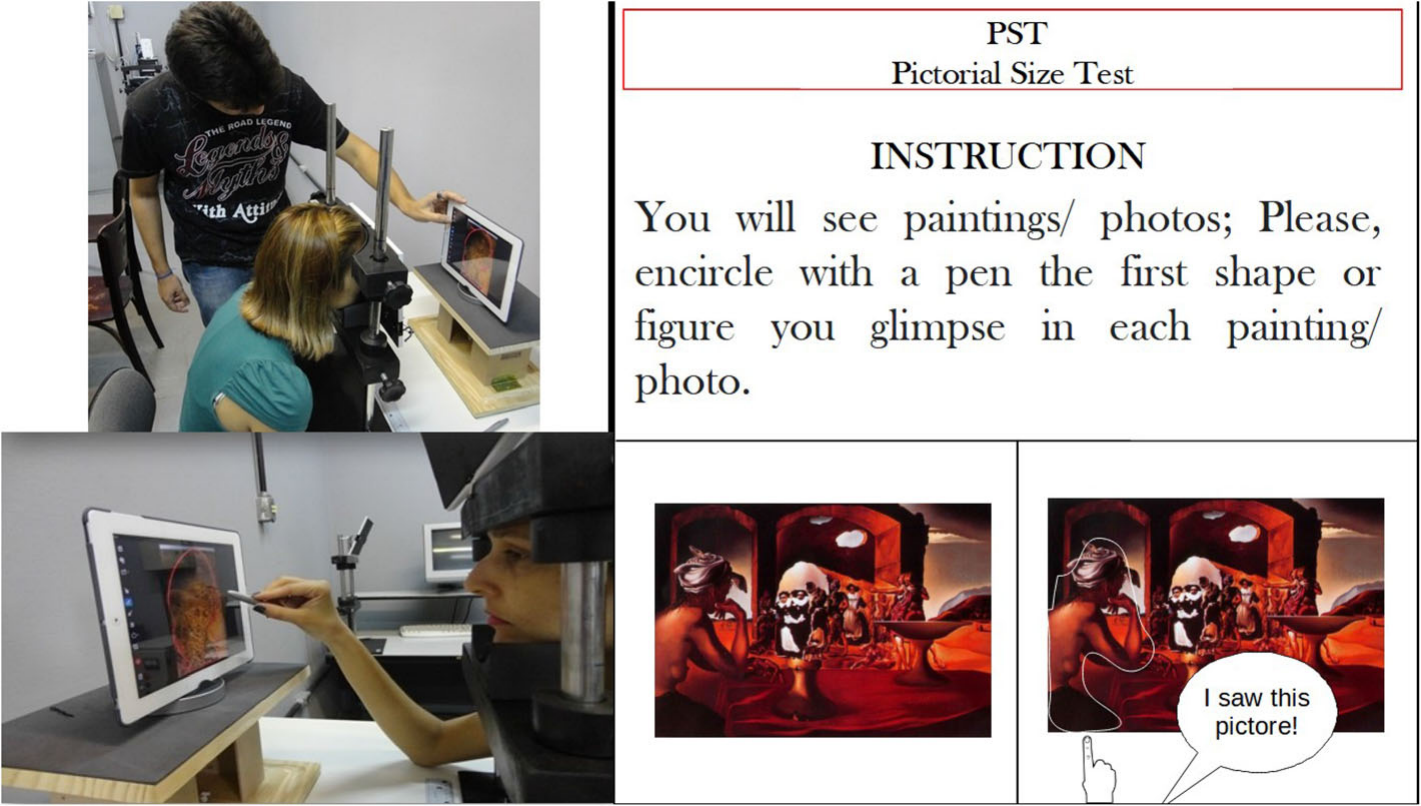
Experimental apparatus and instructions. The actual setting depended on the site where data was being collected

Paintings and plates were presented in an iPad2 display, at 30 cm distance from the eye. The images measured 30 degrees of visual angle in its larger dimension. We also used iPad pens, iPad stands as well as forehead and chin rests (refer to Fig. 2).

Either the app “PDF Notes” or the PowerPoint for the iPad were used to display the stimuli, and the software “Klonk Image Measurement” by Christian Andersen, and “ImageMeterPro” by Dirk Farin, for measuring diameter, perimeter, and area, though only diameter in degrees of visual angle is the focus here.

### Procedure

Volunteer patients were informed about the general aspects of the experiment followed by reading and signing the consent form after fully understanding the research explanation and the task to be performed. After filling a brief form on personal data and being interviewed about familial history, acuity measurements were made at 30 cm distance. Patients with normal (or corrected to normal) visual acuity were then assessed with both MMSE and PANNS. Following the same initial procedure, healthy volunteers were told about the experiment, read and signed the consent form, answered the same interview, run the acuity tests, and were also assessed by the MMSE, but not the PANNS.

As shown in Fig. 2, the volunteer sat with his head at 30 cm from the iPad2 display while resting his forehead and chin on a suitable support. The instruction given was: “You will see some paintings, please encircle with the iPad pen the first at glance image you see”, followed by a single training trial (see Fig. 2). The experiment would start with a block of 10 Dali paintings followed by the block of 10 Rorschach plates (for part of the volunteers – 33 EG and 33 CG) always in the same order. Although there were no time restrictions, the experiment was short and lasted about 10 min on the average. For some in-patients, short intervals were made whenever requested. In case the experiment was run over a period of 2 or 3 days for the in-patients, a short version of the MMSE was run each day previously to the experimental session.

## Results

**Fig. 3:**
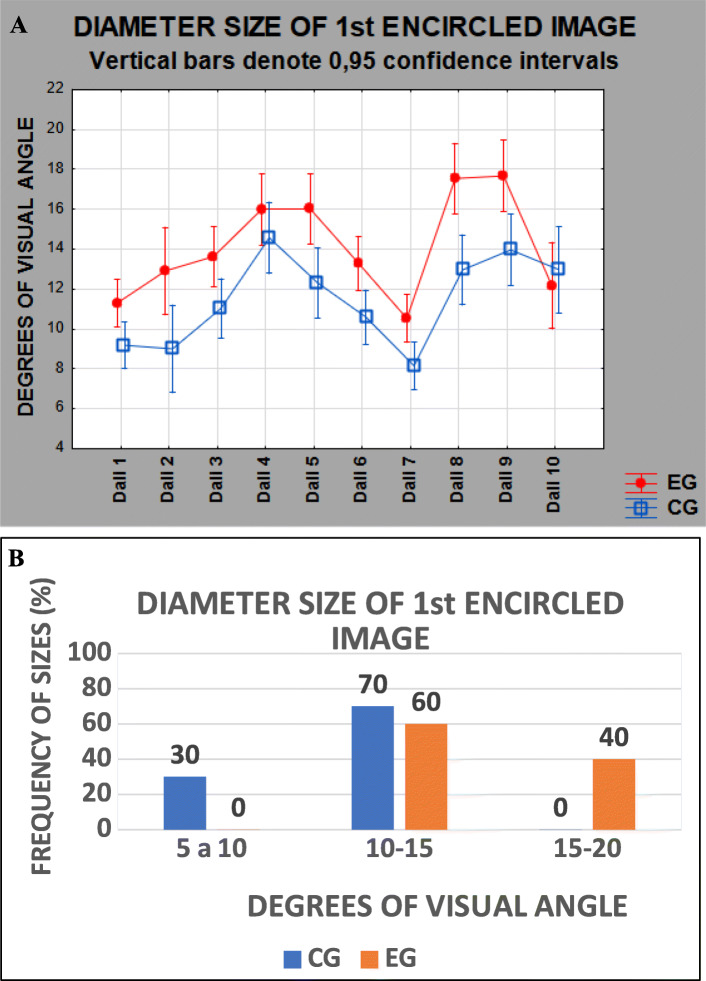
Diameter size of 1st encircled image for Dalí’s paintings by group (**A**). The differences between groups were significant. Frequency of mean sizes, in degrees of visual angle, of the first images seen by volunteers in the 10 paintings of Dalí (**B**). Differences between groups are consistent across stimuli

The diameters of the encircled images were measured in centimeters and converted to degrees of visual angle, then organized by participant, configuration, picture, and group. An ANOVA with one factor between (group: GC vs EG) and one factor within (picture: 10 levels, i.e., paintings) showed a significant difference between groups (*F*_(1, 64)_ = 9.2441, *p* = 0.00342) for Dalí paintings, considering all 118 volunteers. The EG perceived images 1.2-folds greater than CG, thus, showing that in-patients with schizophrenia, even medicated, show a tendency to perceive larger images than people without a known history of neuropsychiatric disease. There was no interaction between groups and pictures (refer to Fig. 3A).

The tendency to perceive larger magnitudes can be seen in Fig. 3B, where the frequency of perceived sizes is distributed by degrees of visual angle. The EG circled images mostly between 10° and 20° of visual angle, while the CG, mostly images between 5° and 15° of visual angle. Only EG circled images greater than 15° of visual angle, and only CG circled images smaller than 10° of visual angle.

**Fig. 4:**
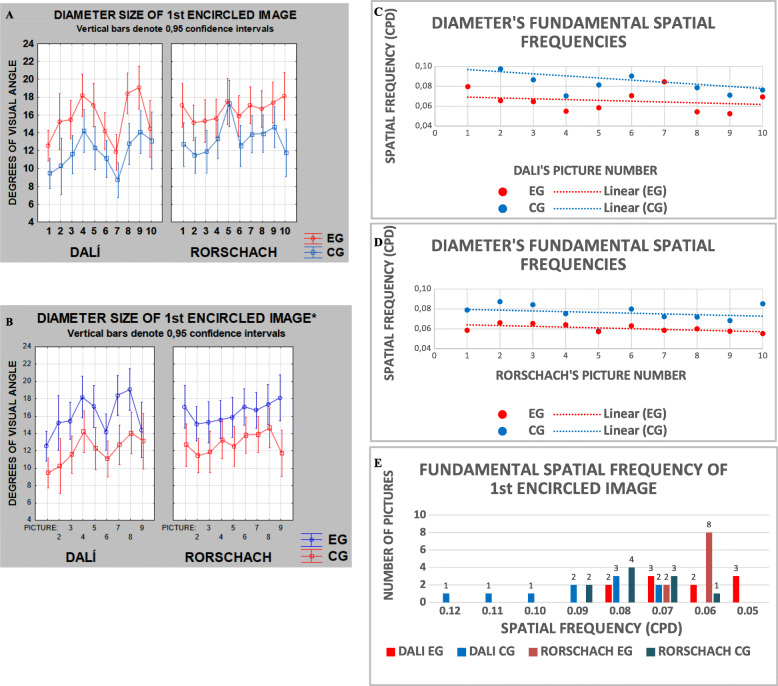
Diameter size of 1st encircled image for Dalí and Rorschach stimuli by group (**A**). Diameter size of 1st encircled image for Dalí without number 7 and Rorschach without number 5 (**B**) for EG and CG. Differences between groups were significant in both cases. Fundamental Spatial Frequencies for both Dalí (**C**) and Rorschach (**D**) pictures. Notice the parallelism of the linear tendency lines (**C**, **D**). Observe that most of the FSF for EG are in the range of 0.06–0.07 cpd, spatial frequency contrast sensitivity values yet to be measured and reported in the literature (**C**, **D**)

Another ANOVA with part of the sample including 66 (33 CG, 33 EG) of the participants compared groups for the two configurations: Rorschach only and Dali paintings vs Rorschach plates, with one factor between groups (GC vs EG) and two factors within groups (configuration: Dalí vs Rorschach, and pictures: 10 paintings vs 10 plates). There was a significant difference between groups (*F*_(1, 64)_ = 6.1027, *p* = 0.01617) in the Rorschach configuration. In this case, EG saw images 1.2 folds greater than CG. When we consider both Dali paintings and Rorschach plates, we also observed a difference between groups (*F*_(1, 64)_ = 8.1236, *p* =0.00587) where the EG saw images 1.3-folds greater than the CG. But, in this case, we found a significant difference between configurations. Such a difference disappears when we subtract from the stimulus set, Rorscharch’s plate number 5 (a bat-like image seen by both groups as the same size, refer to Fig. 4A). For comparison purposes, we arbitrarily subtracted Dalí’s painting number 7 and re-did the analysis.

This new ANOVA (18 pictures and plates set) yielded again significant difference between groups (*F*_(1, 64)_ = 8.9722, *p* = 0.0039), but this time, there was no difference between configurations (*F*_(1, 64)_ = 3.0489, *p* = 0.08559). The interaction between groups and configurations was not significant, showing a similar trend for Dalí’s paintings and Rorschach’s plates in both groups (Fig. 4B).

When we estimated the fundamental spatial frequency (FSF) from the size given in degrees of visual angle (SIZE) by considering that FSF=1/SIZE, we observed quasi-parallel interpolated lines for both, Dalí’s paintings (Fig. 4C) and Rorschach’s plates (Fig. 4D). In doing so, we found that EG volunteers seem to have preferred fundamental spatial frequencies mostly below 0.07 cpd (cycles per degree), while CG volunteers appear to have preferred FSF predominantly above such a value, either for paintings or plates.

In Fig. 4E, we observe this tendency most clearly. The EG (red and orange bars) are mostly below the axis value of 0.07 FSF, while the CG (light and dark blue bars) are mostly distributed above the FSF of 0.09 cpd. This is true even for the complete stimulus set.

## Discussion

As shown in Figs. 3 and 4, EG perceived sizes that were significantly larger than the CG using the digital image on the iPad2 display. Results are consistent with previous studies (Modesto, 2012; Simas et al., 2011) using printed material, and reported in terms of spatial frequencies that found a progressive increase in the perceived size of PLI by schizophrenic patients, as compared to healthy volunteers. We suggest that such an increase in preference for larger sizes may correspond to the degree and severity of the psychotic and/or prodromal states.

**Fig. 5:**
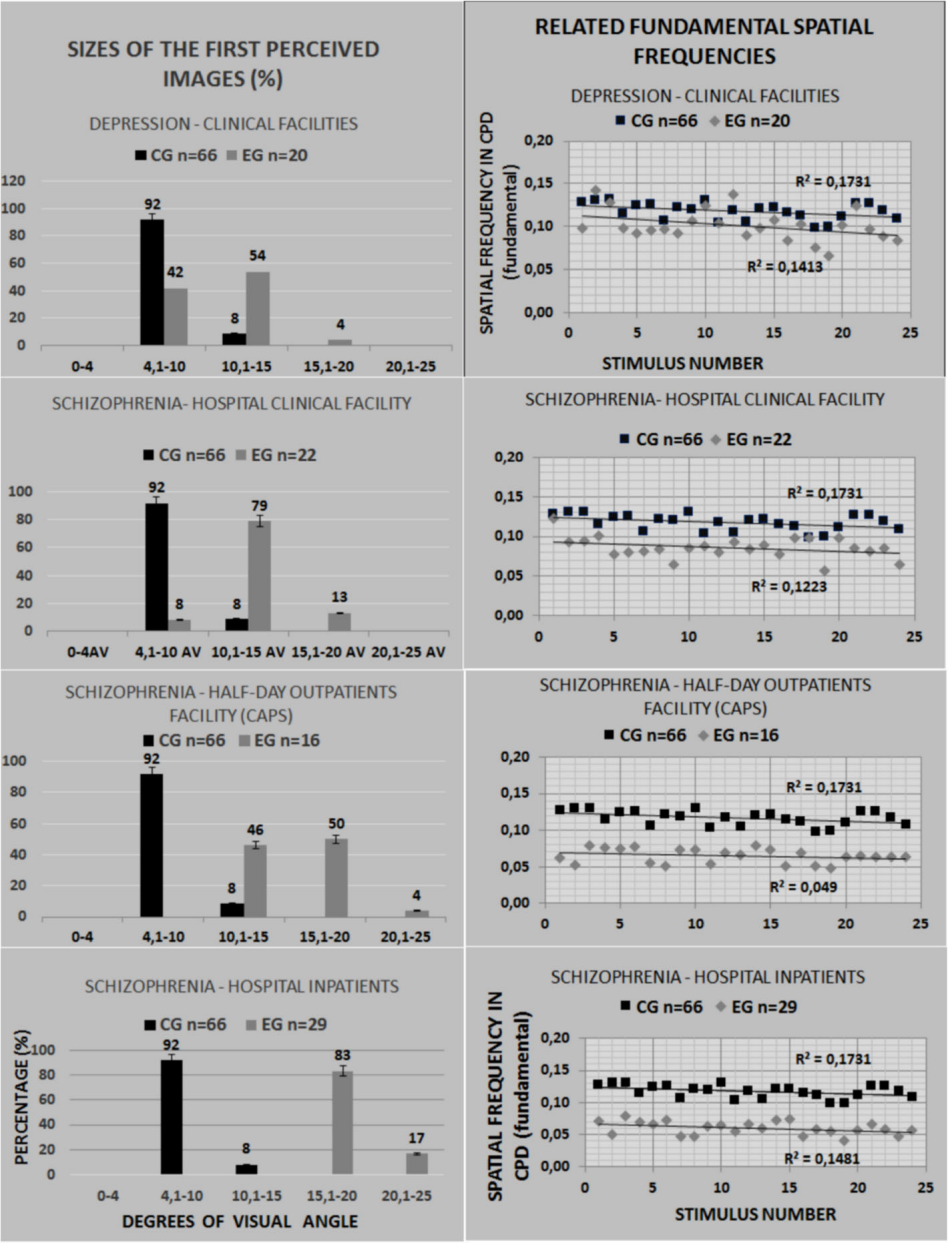
Sizes of the first perceived images from previous studies (Simas, et al., 2011; Lacerda, Simas, & Menezes, 2020; Modesto, 2012 [unpublished M.A. Thesis]) both in degrees of visual angle (left) and in spatial frequency of the respective fundamentals (right). The control groups of three experiments were treated as a single group (*N*=66). *Note.* Original figure including results adapted from Simas et al. (2011), from Lacerda et al. (2020), and from Modesto (2012)

Figure 5 is an adapted summary of previously observed sizes for the first at glance image from three early studies (Modesto, 2012; Simas et al., 2011) using 24 paintings by Dali as printed photographs. It illustrates the fact that controls perceive smaller images than patients with depression who, in turn, perceive smaller images than schizophrenic patients attending a clinic once monthly who, in turn, perceive smaller images than schizophrenic day-clinic patients who, in turn, perceive images smaller than schizophrenic hospital intern patients. Figure 3B shows results from the present experiment in terms of percentage of the observed frequency of sizes per degrees of visual angle for each population made for comparison with Fig. 5. Figures 4 A and B also show data in terms of FSF suggesting that patients perceive spatial frequencies below 0.07 cpd. Figure 4E illustrates this same preference by patients (red and purple bars).

This preference for large magnitudes is consistent with the reported alterations in processing fundamental low spatial frequencies (LSP) (Dias et al., 2011; Graham & Meng, 2011; Kantrowitz et al., 2009; Laprevote et al., 2013). Our studies, however, seem to show increased sensitivity to FSF that would be in the range of 0.075-0.050 cycles per degree (cpd) and out of the commonly investigated range (generally 0.2 cpd, rarely down to 0.1 cpd). In addition, if we hypothesize that sensitivity to brightness in schizophrenic acute or chronic states is abnormally increased, we might expect higher contrast sensitivity at fundamental LSF, as is indeed generally observed. This could be because the visual system is saturating even at low contrast levels (probably a consequence of higher perceived brightness) across the spectrum. This also could explain the finding by Kantrowitz et al. (2009) of poor performance in the illusion of the Herman grid at high contrasts.

Concerning artwork and artwork masterpieces, a study investigating paintings by schizophrenic artists (Graham & Meng, 2011) showed that the images had a high content of LSF, coherent with our findings. Also consonant with that, the perception of hybrid stimuli with high and LSF content, by schizophrenics, rely heavily on the coarse visual information content of LSF. That is, when observing a picture, schizophrenics are using, preferentially, the fundamental LSF information contained within the image, and the image surroundings. This would be equivalent to perceiving large/huge PLI (Cornsweet, 1970). We could go further and argue that some odd and brilliant artwork may derive from such altered perception (as could be the case of some of Salvador Dali’s paintings).

In this context, it is also relevant to the fact that Uchiyama et al. (2012) did not find a correlation between perceiving hallucinations and perceiving pareidolias, though they suggested that those people with DLB that see pareidolia, but do not hallucinate, may be susceptible to hallucinations. In agreement with that observation, our early studies did not show a correlation between the frequency of positive symptoms (assessed by PANNS in the item that included ‘perceiving hallucination’), and the perception of larger PLI. Thus, we conceive our results as measuring a new and distinct kind of phenomenon in schizophrenia that resides heavily on LSF and on disrupted preferred size and/or pictorial perception.

Results from studies with illusions carried out by Weckowicz and Witney (1960), Pessoa et al. (2008), and Shoshina et al. (2011) are in complete agreement with our current and previous findings (Lacerda et al., 2020), all suggesting hypersensitivity to the very low spatial frequency spectrum contained in pictures, objects, and surroundings.

## Conclusion

In the present study, we restricted ourselves to the visual psychophysical evidence related to schizophrenia. Electrophysiological evidence, including the mismatch negativity (MMN), as well as the research on Theta, and Gamma amplitude modulation, do point out to abnormal visual and auditory perceptual processing of stimuli in schizophrenia. It is suggested that top-down cognitive processes do not prevail over bottom-up processes, and that spatiotemporal integration is highly affected in psychosis and schizophrenia as shown, for example, by Basar-Eroglu and colleagues (Rürup et al., 2020).

So, what are the implications of these findings in everyday life in a psychotic afflicted mind under those circumstances? Perception of context seems to be the most affected. This could also implicate in an altered cognition contextualization and interpretation of images and facts. And that indeed happens in schizophrenia.

Thus, considering the present results, as well as the progressive changes we have observed in previous works and presented here, we suggest that the altered pictorial size perception effect in schizophrenia be further investigated, and eventually be used as a tool for the screening of neuropsychiatric patients, because assessing the extent of the alterations may help to evaluate the frequency and severity of schizophrenic, or psychotic, symptoms in early prodromal states.

Please note that, in this work, we dealt mainly with the helm of *the visual spatial domain*. We did not deal with the highly affected *visual, cognitive, and auditory temporal domains.*

Indeed, by taking auditory and temporal domains into account, we should observe that together with the present work, we are also carrying research on hearing tolerance with patients diagnosed with first episode psychosis (FEP), or schizophrenia, or panic attack syndrome, aiming to develop yet another assessment test (to be taken together with the pictorial size test) to help screening and improve general neuropsychiatric diagnosis as well. We already have some encouraging primary results in this ongoing research.

## Data Availability

The datasets generated and/or analyxed during the current study are not publicly available due to the rights of privacy regarding the volunteers for the experiments, but are available from the corresponding author on reasonable request.
